# Green Veterinary Pharmacology Applied to Beekeeping: Semi-Field and Field Tests Against *Varroa destructor*, Using Essential Oil of Bergamot (*Citrus bergamia*) and Lemon (*Citrus limon*)

**DOI:** 10.3390/vetsci12030224

**Published:** 2025-03-02

**Authors:** Roberto Bava, Ernesto Palma, Rosa Maria Bulotta, Stefano Ruga, Giovanna Liguori, Renato Lombardi, Carmine Lupia, Mariangela Marrelli, Giancarlo Statti, Vincenzo Musella, Domenico Britti, Fabio Castagna

**Affiliations:** 1 Department of Health Sciences, University of Catanzaro Magna Græcia, 88100 Catanzaro, Italy; roberto.bava@unicz.it (R.B.); rosamaria.bulotta@gmail.com (R.M.B.); rugast1@gmail.com (S.R.); musella@unicz.it (V.M.); britti@unicz.it (D.B.); fabiocastagna@unicz.it (F.C.); 2 Local Health Autorithy (ASL) Foggia, 71121 Foggia, Italy; giovanna.liguori@aslfg.it (G.L.); renato.lombardi@aslfg.it (R.L.); 3 Mediterranean Ethnobotanical Conservatory, Sersale (CZ), 88054 Catanzaro, Italy; studiolupiacarmine@libero.it; 4 Department of Pharmacy, Health and Nutritional Sciences, University of Calabria, 87036 Cosenza, Italy; mariangela.marrelli@unical.it (M.M.); g.statti@unical.it (G.S.)

**Keywords:** *Varroa destructor*, honeybees, essential oils, semi-field and field study, green veterinary pharmacology

## Abstract

*Varroa destructor* is an ectoparasitic mite that causes a true syndrome with complex symptoms in honeybees. For mite control, several synthetic active principles are commercially available. However, these formulations are proving less effective due to the acquisition of resistance mechanisms by the parasite. Naturally derived compounds, such as essential oils, are less prone to generating resistance mechanisms in mite populations and have been proven effective in numerous independent laboratory investigations. In this study, semi-field and field tests were conducted to evaluate the acaricidal efficacy of two essential oils (*Citrus bergamia* and *Citrus limon*), which, in our previous residual contact toxicity tests, showed efficacy against *Varroa* mites. For semi-field cage tests, two-level cages were set up. Filter paper soaked with different concentrations of essential oils was placed in the lower compartment of the cage; 20 honeybees and 10 *Varroa* mites were allocated in the upper level. Mite detachment from the honeybees was evaluated after 24 and 48 h. For field tests, cardboard strips soaked with solutions of essential oils at different concentrations were inserted in the experimental hives. Weekly, the strips were replaced, and the fall of parasites on the diagnostic bottom board was assessed. Semi-field tests showed that the essential oils, at the concentrations used, were not toxic to bees. In the semi-field tests, an average acaricidal efficacy of 33% and 60% was obtained, at the highest concentration used, for bergamot and lemon essential oils, respectively. During the 4 weeks of exposure to 600 mL of BEO, the average mite mortality was 28.5%. In the experimental group treated with the intermediate concentration of 1200 mL, the average mite mortality at the end of four weeks was 30.2%, while with the highest concentration of 1800 mL, it was 40.3%. During the four weeks of exposure to 600 mL of LEO, the average mite mortality was 34.1%. In the experimental group treated with the 1200 mL concentration, the average mite mortality at the end of the four weeks was 46.6%, while with the highest concentration, it was 50.7%. This study shows that the acaricidal efficacy observed in field tests differs from that in laboratory tests. Innovative formulations are needed that allow the time-controlled and gradual release of essential oil in the field.

## Simple Summary

*Varroa destructor* parasitosis represents the most serious threat to honeybee health. The parasite causes damage to the host, which undermines its survival. Controlling infestation levels through drug treatment becomes a critical process for a colony. In this study, we verified the acaricidal efficacy of two essential oils (*Citrus limon* and *Citrus bergamia*) that, in previous laboratory tests performed by residual contact toxicity tests, have shown good toxicity toward *Varroa*. Bergamot and Lemon essential oils returned, at the highest concentration used in the field, an average percentage of *Varroa* neutralization of 40.3 and 50.7%, respectively. Advanced formulations that allow the gradual and controlled release of essential oil over time are needed to increase acaricidal efficacy.

## 1. Introduction

*Apis mellifera* L. (Hymenoptera: Apidae) is the most important pollinator for agricultural crops worldwide. Its contribution is estimated to be worth billions of USD in the United States alone [1,2,3]. For many farms, ensuring sufficient crop pollination is only possible by utilizing honey bee colony services [4]. However, honey bee management is becoming increasingly challenging due to many factors, including the spread of pests [5,6,7] and the improper and extensive use of pesticides and herbicides [8,9,10]. One of the most critical threats to beekeeping is varroosis, caused by the ectoparasitic mite *Varroa destructor* (Acari: Varroidae). This parasite feeds on the fat body and hemolymph of both adult and immature bees [11,12,13]. Infestation by *V. destructor* weakens the bees’ immune systems, reduces the weight of emerging adults, and alters the chemical composition of the cuticle [14]. Moreover, *V. destructor* serves as a vector for debilitating viruses, including the Israeli acute paralysis virus, Kashmir bee virus, and Deformed Wing virus [14,15]. These effects cumulatively shorten the lifespan of bees, ultimately leading to colony collapse [11]. The control of *V. destructor* in commercial beekeeping relies primarily on the application of synthetic acaricides. While the commercial preparation based on these products is convenient and cost-effective, they have significant drawbacks, including the development of acaricide-resistant mite strains [16,17]. Additionally, their persistence in the environment [11] and possible accumulation in hive products, due to their lipophilic nature, pose ecological and food safety concerns [18]. Eco-friendly alternatives, such as organic acids (e.g., oxalic acid and formic acid) and essential oils (EOs) and/or monoterpenes of EOs show promise. Nevertheless, several studies indicate that using organic acids to counteract *Varroa* could be detrimental to bees. For instance, open and capped broods are more frequently damaged and removed [19,20]. Furthermore, there have been reports of long-term harm to bees’ digestive and excretory organs and glands [21,22], harm to the queen, or, frequently, even early death [23,24], as well as a drop in the pH of honey the next season [25]. Although they require high doses to be effective and can induce swarming in colonies [26,27], at present, EOs appear to be the most harmless and effective compounds for *Varroa* control [28,29]. Several EOs have been tested against *V. destructor*, with varying degrees of success [29,30,31,32]. Most of the studies have been conducted under laboratory conditions; only a limited number have been evaluated under field conditions. This represents an important gap that must be filled to conscientiously assess the efficacy of EOs against *Varroa*. Indeed, field conditions are variable, and the effective concentrations of oils tested in the laboratory may not be sufficient, requiring much higher concentrations. High concentrations could ultimately cause side effects in hives, which makes it necessary to assess EOs in the field. This study aimed to evaluate the effectiveness of *Citrus bergamia* and *Citrus limon* EOs (BEO, LEO) in controlling varroosis directly within hives. The investigation involved a two-step process: first, assessing the oils’ ability to induce *V. destructor* detachment from its host and their toxicity to *A. mellifera* in a laboratory setting; and, second, testing their efficacy in an experimental apiary.

## 2. Materials and Methods

### 2.1. Essential Oils’ Extraction and Characterization

Citrus fruits, cultivated and collected in the province of Reggio Calabria (Southern Italy), were carefully peeled to separate the outer rind from the pulp. The collected peels were then finely chopped and minced to increase the surface area and facilitate the extraction process. The pre-processed peels were subsequently transferred into a hot oil bath and subjected to vacuum distillation, a technique designed to extract essential oils (EOs) at reduced temperatures, thereby preserving their volatile compounds and minimizing thermal degradation. During this process, the volatile components were carried by steam, leading to the separation of the essential oil from the plant matrix. Following distillation, the condensate was collected and separated into an aqueous phase and an essential oil phase. To obtain a concentrated product, the essential oil was carefully extracted from the aqueous phase and subjected to a drying process to remove residual moisture. This final step ensured the purity and stability of the extracted EO before further testing and application. Tested samples were analyzed using gas chromatography–mass spectrometry (GC-MS) with a Hewlett–Packard 6890 gas chromatograph connected to a Hewlett–Packard 5973 mass spectrometer (Agilent, Milan, Italy). The system was equipped with an SE-30 capillary column made entirely of dimethylpolysiloxane (30 m in length, 0.25 mm in diameter, and with a 0.25 µm film thickness). Analyses were conducted with helium as the carrier gas (linear velocity of 0.00167 cm/s) and a programmed temperature range of 60 to 280 °C at a rate of 16 °C/min. The EO components were identified by comparing the retention times with those in the Wiley 138 mass spectral library [33].

### 2.2. Varroa Mite and Honeybees Collection for Semi-Field Tests

*V. destructor* and honeybees were collected from an apiary in the Catanzaro province, Calabria, Italy. The collection period was late spring. A brood comb, which had been placed in the hive four weeks before the start of the experiment, was used. Specifically, four weeks before the commencement of the toxicological tests, a drone frame was introduced into the hive. Once most of the brood was capped, the frame was transferred to the laboratory, where each brood cell was inspected for *Varroa* mites. Each larva or pupa was removed from its cell, and the cell was thoroughly examined. When present, the mites were collected with a fine paintbrush and placed in a Petri dish along with a fifth-instar larva or pupa as food [34]. The honeybees were obtained from brood combs removed from healthy colonies and incubated in the laboratory to allow them to emerge.

### 2.3. Semi-Field Tests

Two-level cylindrical cages, each 10 cm in height and 9 cm in diameter, were prepared to evaluate the effectiveness of EO vapors, following a similar method to that described by Bava et al., 2022 [34]. The two floors of the cage were physically separate and differed in their contents. The upper portion housed 10 mites and 20 adult honeybees. The honeybees were derived from brood combs, removed from healthy colonies, and incubated in the laboratory to allow them to emerge. Once emerged, the young honeybees were allocated inside the cage and provided with 50% sugar syrup, available ad libitum. A filter paper soaked in EO solutions (distilled water mixed with EO at different concentrations and an emulsifier) was placed in the lower section of the cage. Specifically, 2 × 2 cm strips of Whatman No. 1 filter paper were soaked using different EO concentrations. The treated filter paper was placed in the lowest section of the fumigation chamber. Four different doses were tested. The Whatman filter paper was treated with 1 mL of an aqueous solution of *Citrus bergamia* and *Citrus limon* EOs, with concentrations ranging from 0% to 4%. Each concentration was tested in five replicates. For the separation of mites from the host to be considered valid, the adult honeybees had to remain alive at the end of the test. Abnormal bee behaviors such as disorientation, motor incoordination, and apathy were observed one hour after treatment and assessed again at 24 and 48 h later. Mite mortality was also assessed at 24 and 48 h.

### 2.4. Field Tests

For the execution of field tests, the methods implemented by Conti et al. (2020) [28] were followed. In September 2023, bioassays were carried out at a beekeeping farm located in the province of Catanzaro, Calabria, Italy. The average daily temperature ranged between 16 and 28 °C, with a relative humidity (RH) of approximately 70%. The apiary consisted of 30 hives, equally managed and balanced in strength, population size, brood quantity, and food resources. The number of sealed brood cells in each selected colony was comparable, estimated to be no more than one frame.

According to Conti et al. (2020) [28], plywood sticks (4–20 cm) were used to administer the EOs to the hives. Each stick was previously treated with 2 mL of ethanol solutions containing 0 (control), 600.0, 1200.0, or 1800.0 mL of *C. bergamia* and *C. limon* EOs. After the ethanol evaporated completely in a fume hood, the sticks were wrapped in aluminum foil and stored at −20 °C until use. Two treated sticks (40 × 40 × 50 = 80,000 cm^3^) were placed in each hive, suspended between the frames using a toothpick. Of the 30 hives in the experimental apiary, 5 hives were treated with the lowest concentration of EO, 5 with the intermediate concentration, and 5 with the highest concentration.

Trays coated with baking paper sprayed with mineral oil were placed at the bottom of the hives to collect fallen *V. destructor*. Every seven days throughout the experiment, the treatment was stopped due to the volatility of the OEs, the fallen mites were counted, new trays lined with baking paper were inserted, and new strips imbibed with essential oils were inserted. Each treatment was performed four times.

At the end of the experimental period, a final follow-up treatment was applied according to the guidelines of the European Working Group for the coordination of research on integrated *Varroa* control (Commission of the European Communities, 2002) [28]. This involved a double dose of oxalic acid (*Apibioxal* VR, Chemicals Laif, SpA, Vigonza, PD, Italy) to kill and count any remaining mites. To account for potential mite resistance to synthetic acaricides [35,36] and minimize the overestimation of EO treatment efficacy, the double oxalic acid dosage was recommended. We included control groups in order to not overestimate the results obtained with EO treatments and to correct fall data with mortality and natural mite fall determined by natural host defense systems (e.g., grooming behavior, varroa-sensitive hygienic activity, etc.). The control groups were subjected to weekly counts of mites that had fallen on the trays, and at the end of the experiments, received the same follow-up treatment. The following formula was used to determine the effectiveness of the EO treatments:

D% = 100 × (A − A × B/100)/(A + C);

D% = the relative percentage of mites that fell off *V. destructor*;

A = the number of *V. destructor* mites found on the tray following the EO treatments;

B = the percentage of *V. destructor* mites falling off in the control treatments;

C = the number of *V. destructor* mites falling off following the follow-up treatment.

### 2.5. Statistical Analysis

Statistical analysis was performed using GraphPad PRISM (version 10, GraphPad Software Inc., La Jolla, CA, USA). To assess the normality of the data, we applied the Shapiro–Wilk test, which tested the null hypothesis that the data would follow a normal distribution. When normality was confirmed, comparisons between different treatment doses were made using repeated-measures ANOVA, which accounted for the correlation between measurements taken from the same subject over time. For data that did not meet the assumption of normality, non-parametric tests were applied. Specifically, the Mann–Whitney U test was used to compare data between two independent groups, providing a robust method for non-normally distributed data, involving dose and exposure time. All results are presented as means ± standard errors of the means (SEMs). Statistical significance was determined based on a *p*-value threshold of ≤0.05, indicating a significant difference between the groups tested.

## 3. Results

### 3.1. Chemical Profile

Table 1 shows that at least 11 compounds were found in the bergamot essential oil (BEO). Both EOs had the following in common: thujene, alpha-pinene, camphene, beta-pinene, beta-myrcene, limonene, and linalool. The main chemical represented class was monoterpene hydrocarbons. In every sample that was examined, limonene was the most prevalent component. Only the lemon essential oil (LEO) sample contained phellandrene, gamma-terpinene, alpha-terpinolene, cis-carveol, and citral. Unlike the lemon, the bergamot contained linalool oxide, p-mentha-2,8-dien-1-ol, trans-carveol, and linalyl acetate.

### 3.2. Semi-Field Tests

**Figure 1:**
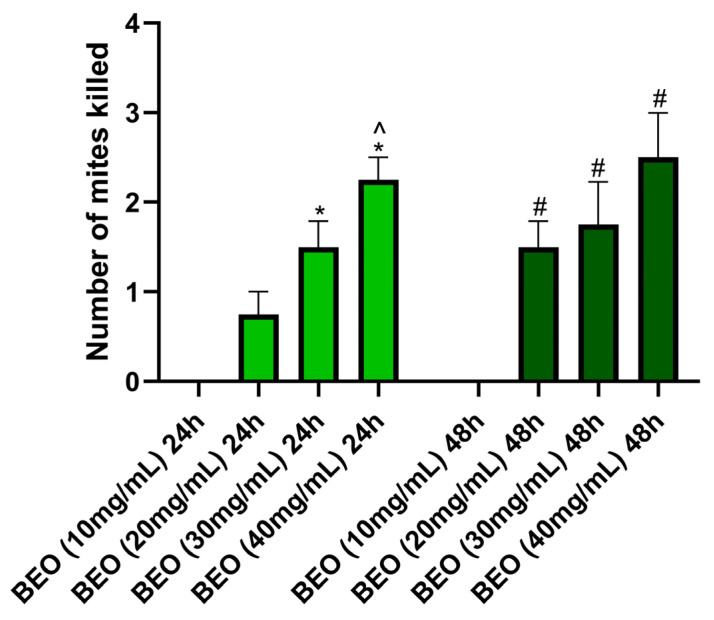
Effects of BEO on neutralization of mites. * *p* < 0.05 vs. BEO (10 mg/mL) after 24 h; # *p* < 0.05 vs. BEO (10 mg/mL) after 48 h; ^ *p* < 0.05 vs. BEO (30 mg/mL) after 24 h.

BEO at the concentrations of 20 mg/mL, 30 mg/mL, and 40 mg/mL was significantly more effective in neutralizing mites after 24 h of exposure than that at the concentration of 10 mg/mL at the same time of exposure (*p* < 0.05). Similarly, BEO concentrations of 20 mg/mL, 30 mg/mL, and 40 mg/mL produced a higher significance of efficacy than BEO at 10 mg/mL after 48 h of exposure (*p* < 0.05). BEO with a concentration of 40 mg/mL produced significantly more mite deaths after 24 h of exposure than BEO at 30 mg/mL during the same hours (*p* < 0.05) (Figure 1). However, no statistically significant difference was found between BEO at 30 mg/mL and BEO 40 at mg/mL after 48 h of exposure. At the maximum exposure time, the average percentage of mites neutralized by BEO was 0% at a concentration of 10 mg/mL, 16% at 20 mg/mL, 26% at 30 mg/mL, and 33% at 40 mg/mL.

**Figure 2:**
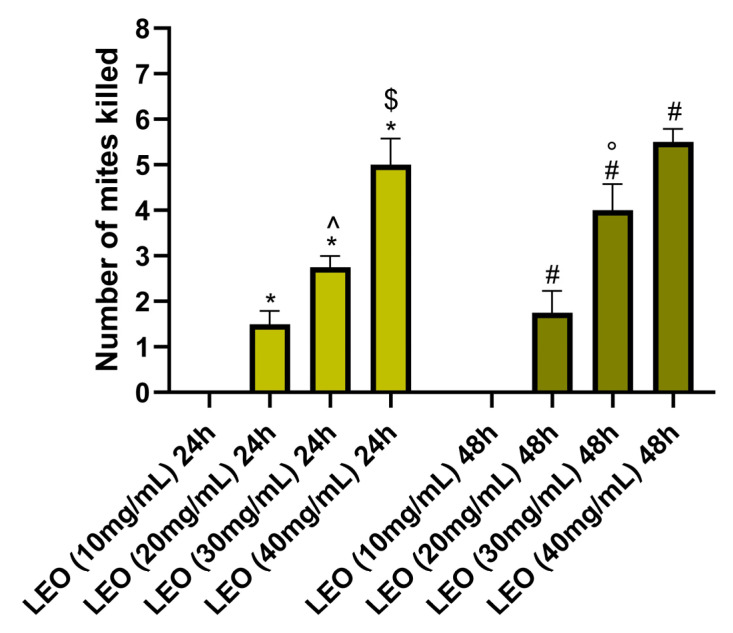
Effects of LEO on neutralization of mites. * *p* < 0.05 vs. LEO (10 mg/mL) after 24 h; # *p* < 0.05 vs. LEO (10 mg/mL) after 48 h; ^ *p* < 0.05 vs. LEO (20 mg/mL) after 24 h; ° *p* < 0.05 vs. LEO (20 mg/mL) after 48 h; $ *p* < 0.05 vs. LEO (30 mg/mL) after 24 h.

LEO at the concentrations of 20 mg/mL, 30 mg/mL, and 40 mg/mL was significantly more effective in neutralizing mites after 24 h of exposure than that at the concentration of 10 mg/mL at the same time of exposure (*p* < 0.05). Similarly, LEO concentrations of 20 mg/mL, 30 mg/mL, and 40 mg/mL produced a higher significance of efficacy than LEO at 10 mg/mL after 48 h of exposure (*p* < 0.05). LEO at the concentration of 30 mg/mL produced significantly more mite deaths after 24 h of exposure than LEO at 20 mg/mL during the same hours. Even after 48 h, BEO at 30 mg/mL was statistically more significant in neutralizing mites than the 20 mg/mL LEO concentration. LEO at 40 mg/mL caused statistically significantly more mites’ deaths than LEO 30 mg/mL after 24 h of exposure (Figure 2). However, no statistically significant difference was found between LEO at 30 mg/mL and LEO at 40 mg/mL after 48 h of exposure. At the maximum exposure time, the average percentage of neutralized mites by LEO was 0% at a concentration of 10 mg/mL, 18% at 20 mg/mL, 42% at 30 mg/mL, and 60% at 40 mg/mL.

**Figure 3:**
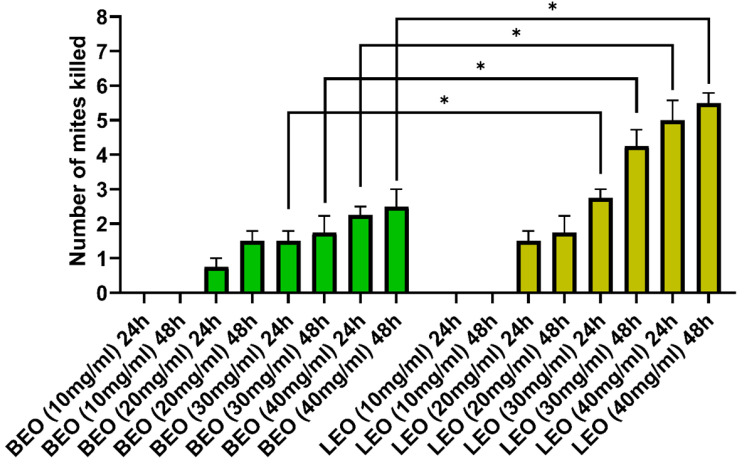
Comparison of effectiveness between BEO and LEO. * *p* < 0.05.

After 24 and 48 h of exposure to BEO and LEO at 10 mg/mL, there was no difference in the number of neutralized mites. The 20 mg/mL concentration of BEO was not significantly significant when compared to the same concentration of LEO. In contrast, LEO at 30 mg/mL and 40 mg/mL concentrations showed a significant increase in the number of dead mites when compared to the same concentrations of BEO after both 24 and 48 h (Figure 3).

Table 2 shows the values of lethal dose 10 (LD10) and 50 (LD50) of the two EOs.

### 3.3. Honeybee Toxicity in Semi-Field Tests

**Figure 4:**
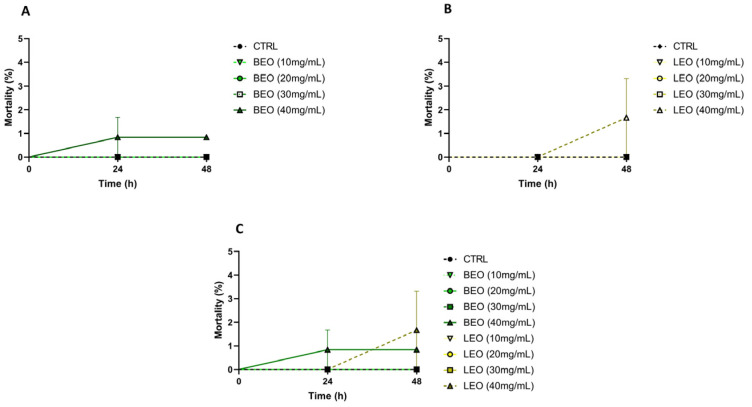
(**A**) BEO and (**B**) LEO mortality rate; (**C**) mortality comparison for BEO and LEO essential oils.

After 24 and 48 h of exposure to BEO at 10 mg/mL, BEO at 20 mg/mL, and BEO 30 at mg/mL, mortality was 0%; with BEO at 40 mg/mL, it was 0.84%. Neither after 24 h nor after 48 h was the mortality statistically significant compared to the control (Figure 4A).

After 24 h of exposure to LEO at 10 mg/mL, LEO at 20 mg/mL, and LEO 30 at mg/mL, mortality was 0%. After 48 h of exposure to LEO at 10 mg/mL, LEO at 20 mg/mL, and LEO at30 mg/mL, it was 0%; LEO at 40 mg/mL returned a mortality percentage of 1.6%. Neither after 24 h nor after 48 h was the mortality statistically significant compared to the control (Figure 4B).

The mortality rate for the different concentrations of BEO and LEO at different exposure intervals (24 and 48 h) showed no significant difference (Figure 4C).

### 3.4. Field Tests

**Figure 5:**
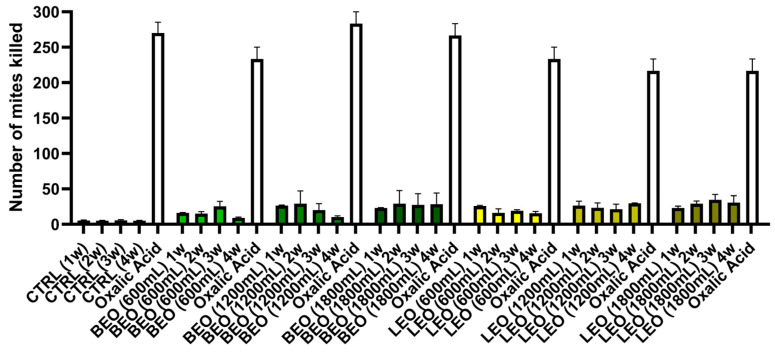
Comparison of effectiveness between BEO and LEO.

During the 4 weeks of exposure to BEO 600 mL, there was an average percentage of mite deaths of 28.5%. In the experimental group treated with the intermediate concentration of 1200 mL, the average mite mortality at the end of the four weeks with 1200 mL of BEO was 30.2%, while with the highest concentration of 1800 mL, it was 40.3%. During the four weeks of exposure to 600 mL of LEO, there was an average percentage of dead mites of 34.1%. In the experimental group treated with the 1200 mL concentration, the average mite mortality at the end of the four weeks was 46.6%, while with the highest concentration, it was 50.7%. The different concentrations of BEO during the four-week exposure produced comparable numbers of mite deaths at the same concentrations and exposure times as LEO. Thus, there was no statistically significant difference between the two EOs (Figure 5).

Table 3 shows the values of lethal dose 10 (LD10) and 50 (LD50) of the two EOs.

## 4. Discussion

*V. destructor* parasitosis is a critical issue for beekeeping worldwide, requiring extensive research to develop effective control strategies. One promising avenue of research focuses on selecting genetically resistant bee populations that exhibit mite recognition behaviors, self-grooming, allo-grooming and *Varroa*-sensitive hygiene traits [37]. However, this approach requires a long selection period, making immediate interventions necessary. Currently, drug treatments remain indispensable for controlling infestations. Treatments based on synthetic compounds have shown decreasing effectiveness due to the rapid development of resistance in mite populations [17,38,39]. Furthermore, these chemicals persist in the environment and can accumulate in hive products, particularly in lipophilic matrices such as wax [26]. Conversely, treatments based on EOs present a more sustainable alternative. In fact, EOs degrade easily in the environment, and their complex chemical composition makes it difficult for mites to develop resistance mechanisms.

Numerous studies have explored EO-based treatments for *V. destructor*, employing various bioassay techniques, including residual contact toxicity tests, direct contact tests with treated surfaces, and fumigation tests in closed and open chambers [32,40]. However, few studies have assessed their efficacy under field conditions. In this study, we evaluated the field acaricidal efficacy of two EOs, *C. bergamia* and *C. limon*, which showed promising results in our previous residual contact toxicity tests [41]. According to chemical analyses in the literature, the primary components of these EOs include limonene, linalool, α-pinene, β-pinene, and myrcene [42,43]. The observed efficacy against other mite species suggests that these constituents may contribute to the acaricidal properties of the oils [44,45]. In our semi-field tests, both EOs demonstrated moderate acaricidal effects inducing *V. destructor* detachment from *A. mellifera*. However, the neutralization rates were lower than those obtained in previous in vitro contact toxicity tests [41]. Importantly, the tested EOs did not show significant toxicity to honeybees at the administered doses, as bee mortality rates did not differ significantly from those in the control group. Based on these findings, we derived the EO concentrations for the field tests from the semi-field study.

As pointed out in one of our previous publications [34], our semi-field cages had a 1 L air volume, whereas a Dadant–Blatt hive has approximately 60 L of air volume. To achieve comparable saturation levels, we multiplied the semi-field test concentrations by 60, resulting in the field concentrations used of 600 mg/mL, 1200 mg/mL, and 1800 mg/mL. The maximum concentration was selected based on the semi-field results, where no significant difference in *Varroa* drop was observed between the 30 and 40 mg/mL concentrations. This threshold served as a cut-off, preventing further increases in concentration. The acaricide efficacy reported for bergamot and lemon EOs is likely to be that found with the semi-field studies at the highest concentration used. Raising the concentration further would most likely lead to acaricide efficacy results similar to those recorded. In the field study, our concentrations did not differ much from those used by Conti et al. (2020) [28]. Our findings showed that the EOs’ toxicity proved in in vitro toxicity tests [41] against *V. destructor* was not maintained in the toxicological semi-field and field tests. The semi-field study already indicated a decline in efficacy compared to residual contact toxicity tests, and in the field study, acaricidal efficacy decreased further.

Given the substantial differences between test environments (closed laboratory systems versus open field settings with worker bee ventilation, etc.), this discrepancy was expected. One possible explanation for reduced efficacy in the field is the presence of sealed broods, which limited EO penetration into the hives. To mitigate efficacy losses when transitioning from controlled to open systems, future research should focus on incorporating EO into pharmacological carriers that enhance stability and controlled release. One promising technique is encapsulation, where an active compound is enclosed within a protective matrix until triggered by environmental conditions. Various encapsulation methods exist, though many were initially developed for food and pharmaceutical applications rather than biopesticides. Cost-effective techniques such as simple coacervation, using polymers like gelatin or ethyl cellulose, offer a viable approach [46]. Using cyclodextrins (CDs) is another effective strategy. Six, seven, or eight glucose units are cyclically connected to form CDs, also known as α-, β-, and γ-CDs. Foods, cosmetics, toiletries, and medicinal applications all make substantial use of CD complexation. CDs can be thought of as nanoencapsulation agents since they cause complex formation that is comparable to molecular encapsulation. After being separated from one another, bioactive EO molecules are molecularly distributed within an oligosaccharide matrix. Moreover, polymers and EOs can be combined to create sheets. In order to manage insects in horticulture and agriculture, appealing adhesive films containing essential smells have been produced (Klerk’s Plastic Industries B.V., 1990) [47]. The Food and Drug Administration (FDA) and Environmental Protection Agency (EPA) in the United States have fully authorized a large number of commercial products that contain EOs for use in food and beverage preparation as “Generally Recognized as Safe” (GRAS) items, which is encouraging in this regard [48].

## 5. Conclusions

The results of our study confirm that natural acaricide treatments, particularly those based on essential oils, show promise as an eco-friendly alternative to synthetic chemicals in beekeeping. While laboratory studies have demonstrated their efficacy, our findings underline the importance of conducting further field trials to assess the performance of these natural substances in real-world conditions, where environmental factors can impact their effectiveness. Given the increasing resistance of *V. destructor* to conventional treatments, the development of new formulations that allow for the controlled and sustained release of active compounds is essential. This approach would reduce the frequency of interventions while maximizing efficacy and ensuring environmental sustainability. As such, continued research in this area is vital for supporting the survival of the beekeeping industry and promoting both environmental health and consumer safety from a One Health perspective.

## Figures and Tables

**Table 1:** Chemical characterization of EOs used.

N.	Compound ^(a)^	Rt ^(b)^		RAP ^(c)^
			* Citrus limon *	* Citrus bergamia *
1	Thujene	6.197	2.22	1.05
2	Alpha-pinene	6.363	7.97	3.21
3	Camphene	6.666	0.49	0.14
4	Beta-pinene	7.254	19.31	9.61
5	Sabinene	7.266	-	-
6	Beta-myrcene	7.517	4.50	2.18
7	Phellandrene	7.746	0.25	-
8	Limonene	8.169	35.21	25.17
9	Gamma-terpinene	8.712	8.39	-
10	Linalool oxide	8.838	-	1.79
11	Alpha-terpinolene	9.049	0.58	-
12	Linalool	9.209	0.26	11.32
13	P-mentha-2,8-dien-1-ol	9.495	-	0.30
14	Lemongrass	9.815	-	-
15	Cis-carveol	10.615	0.32	-
16	Trans-carveol	10.792	-	0.86
17	Linalyl acetate	10.918	-	13.50
18	Citral	11.089	4.14	-
19	Geranyl acetate	12.084	-	-
20	Trans-caryophillene	12.558	Tr ^(d)^	-
21	Uroterpenol	12.680	Tr	-
22	Valencene	13.147	-	-
23	Nerolidol	13.593	-	-
24	Beta-bisabolene	16.502	-	-
25	Neoisolongifolene-8,9-dehydro	18.108	-	-

^(a)^: Compounds listed in order of elution from SE30 MS column. ^(b)^: Retention time (min). ^(c)^: Relative area percentage. ^(d)^: Tr (value lower than 0.1% are reported as traces).

**Table 2:** Lethal dose 10 and 50 of BEO and LEO.

EOs	BEO				LEO			
Concentration	10 mg/mL	20 mg/mL	30 mg/mL	40 mg/mL	10 mg/mL	20 mg/mL	30 mg/mL	40 mg/mL
LD10	0	12.5	11.53	12.12	0	11.11	7.14	6.66
LD50	0	62.5	57.69	60.60	0	55.55	35.71	33.33

**Table 3:** Lethal dose 10 and 50 of BEO and LEO.

EOs	BEO			LEO		
Concentration	600 mL	1200 mL	1800 mL	600 mL	1200 mL	1800 mL
LD10	210.52	397.35	446.65	175.95	257.51	355.02
LD50	1052.63	1986.75	2233.25	879.76	1287.55	1775.14

## Data Availability

Data are available upon request from the corresponding author.
